# Metabolomic Profiles of Men and Women Ischemic Stroke Patients

**DOI:** 10.3390/diagnostics11101786

**Published:** 2021-09-28

**Authors:** Nicolas Poupore, Renee Chosed, Sergio Arce, Robert Rainer, Richard L. Goodwin, Thomas I. Nathaniel

**Affiliations:** 1 School of Medicine Greenville, University of South Carolina, Greenville, SC 29605, USA; npoupore@email.sc.edu (N.P.); chosed@greenvillemed.sc.edu (R.C.); sarceuss@gmail.com (S.A.); GOODWIRL@greenvillemed.sc.edu (R.L.G.); 2 PRISM Health Upstate, Greenville, SC 29605, USA; Robert.Rainer@prismahealth.org

**Keywords:** ischemic stroke, metabolomics, male patients, female patients

## Abstract

Background: Stroke is known to affect both men and women; however, incidence and outcomes differ between them. Therefore, the discovery of novel, sex-specific, blood-based biomarkers for acute ischemic stroke (AIS) patients has the potential to enhance the understanding of the etiology of this deadly disease in the content of sex. The objective of this study was to identify serum metabolites associated with male and female AIS patients. Methods: Metabolites were measured with the use of untargeted, reverse-phase ultra-performance liquid chromatography-tandem mass spectrometry quantification from blood specimens collected from AIS patients. Samples were collected from 36 patients comprising each of 18 men and women with matched controls. Metabolic pathway analysis and principal component analysis (PCA) was used to differentiate metabolite profiles for male and female AIS patients from the control, while logistic regression was used to determine differences in metabolites between male and female AIS patients. Results: In female AIS patients, 14 distinct altered metabolic pathways and 49 corresponding metabolites were identified, while 39 metabolites and 5 metabolic pathways were identified in male patients. Metabolites that are predictive of ischemic stroke in female patients were 1-(1-enyl-palmitoyl)-2-arachidonoyl-GPC (P-16:0/20:4) (AUC = 0.914, 0.765–1.000), 1-(1-enyl-palmitoyl)-2-palmitoyl-GPC (P-16:0/16:0) (AUC = 0.840, 0.656–1.000), and 5,6-dihydrouracil (P-16:0/20:2) (AUC = 0.815, 0.601–1.000). Significant metabolites that were predictive of stroke in male patients were 5alpha-androstan-3alpha,17beta-diol disulfate (AUC = 0.951, 0.857–1.000), alpha-hydroxyisocaproate (AUC = 0.938, 0.832–1.000), threonate (AUC = 0.877, 0.716–1.000), and bilirubin (AUC = 0.817, 0.746–1.000). Conclusions: In the current study, the untargeted serum metabolomics platform identified multiple pathways and metabolites associated with male and female AIS patients. Further research is necessary to characterize how these metabolites are associated with the pathophysiology in male and female AIS patients.

## 1. Introduction

Stroke presents a more significant health burden on women than in men as women present with more stroke events and are less likely to recover [1]. While age-specific stroke rates are higher in men, women present with more stroke incidents than men because of their longer life expectancy and much of a higher incidence at older ages [2]. Similar to age-adjusted mortality, women present with more overall lower age-adjusted stroke incidence than men [3,4]. In addition, functional outcomes and quality of life after stroke are consistently poorer in women, and women are reported to present with worse pre-stroke disability than men [3,5]. Moreover, men and women with stroke differ concerning the prevalence of stroke risk factors [6,7]. Women with stroke are older at onset and are more likely to have atrial fibrillation and hypertension [8]. In contrast, men with stroke are more likely to present with a history of heart disease, myocardial infarction, peripheral arterial disease, diabetes, and alcohol and tobacco use [9,10]. The most common biological explanation for differences in stroke between men and women is posited to be hormonal [11]. In addition, studies in animal models demonstrate that women have smaller stroke volumes than men [12].

The human blood contains a wide variety of chemically diverse low molecular weight compounds, the metabolome, which can be measured in parallel through modern metabolomic technologies [13]. Analysis of the metabolome in AIS patients provides new opportunities to understand the pathophysiology of ischemic stroke [14]. Therefore, metabolomics is a promising technique for the evaluation of global metabolic changes in stroke [13]. By comparing metabolic profiles and their dynamic changes, changes in pretreated and treated patients can be elucidated. Metabolites in blood have been reported to be associated with vascular diseases [15]. For example, lipoprotein associated phospholipase A2 (Lp-PLA2) was reported to be linked with atherosclerosis and transient ischemic attack [16,17], and lysophosphatidylcholines (LysoPCs) were significantly associated with recurrent stroke [18]. In addition, metabolic profiles in AIS are reported to be significantly different from healthy people [19,20]. For example, changes in sphingomyelin and phosphatidylcholine metabolism were independently linked with risk of infarction in healthy adults. While different, there are multiple blood-based biomarkers investigated in AIS patients. For example, multiple blood biomarkers (N-terminal pro-brain natriuretic peptide [NT-proBNP], d-dimer, S100β, neuron-specific enolase, vitamin D, cortisol, interleukin-6, insulin, uric acid, and albumin) were effective in the identification of patients with increased possibility of cardioembolism and AF [21]. Moreover, various blood-based biomarkers including BNP/NT-proBNP, d-dimer, CRP, TNF-α, IL-6, and IL-1 are reported to be significantly associated with ischemic stroke [21,22,23]. Findings from the existing studies indicate that metabolomics is a powerful tool that can be used to explore biomarkers and related pathways in stroke. 

The vulnerability to several diseases and the response to treatments differ between men and women [24]. Therefore, the differential treatment for men and women AIS patients would represent personalized medicine approach [25] to provide care for AIS patients. However, this requires an extensive understanding not only clinical risk factors but also the intrinsic molecular differences between men and women AIS patients. As a basis for a gender-specific care for AIS patients, the characterization of the molecular differences between the men and women AIS patients is necessary. Moreover, affected pathways may reveal gender-specific susceptibility. Knowledge of the underlying metabolic differences might lead to concrete starting points for a future research to improve care for men and women AIS patients. Existing studies [13,14,26,27,28,29,30,31,32,33,34] that investigated metabolites in stroke focused on differences between stroke and control, hemorrhagic strokes compared with non-hemorrhagic or ischemic strokes. For example, elevated plasma DNA concentrations were detected in patients with hemorrhagic strokes compared with non-hemorrhagic strokes, with a 31% sensitivity and 83% specificity for discriminating the two types of stroke [34]. Moreover, plasma levels of miR-124-3p, miR-125b-5p, and miR-192-5p, were found to be elevated and correlated positively with infarct volume of stroke patients [35,36,37]. In addition, protein biomarkers for brain injury and thrombosis were categorized and used to discriminate hemorrhagic stroke from ischemic stroke patients [38]. A recent study by Daokun et al. [13] analyzed serum metabolites and risk of ischemic stroke in men and women patients. The results reveal circulating biomarkers for stroke and novel pathways for AIS and its subtypes. However, differences between men and women patients were not reported. While findings from existing studies [13,14,26,27,28,29,30,31,32,33,34] highlight the potential of metabolomics for discovering novel circulating biomarkers for stroke and its subtypes, existing studies did not report gender-differentiated results. Therefore, the specific metabolites and related pathways that are directly associated with men and women AIS patients are not fully understood. Therefore, we conducted an untargeted metabolomics study to investigate whether metabolic profiles and related pathways are different in men and women patients with AIS.

## 2. Methods

Approval for this study was obtained from the PRISMA Health Institutional review board (Pro00072801), and all methods were performed in accordance with the relevant guidelines and regulations. A total of 36 ischemic stroke patients comprising each of 18 female and male ischemic stroke subjects with matched controls were recruited for this study. Healthy controls were randomly selected from PRISMA Health system. Samples were collected from patients within 24 h of symptom onset based on relevant ischemic lesions on CT or brain MRI. The general criteria for inclusion were as follows: at least 18 years of age and meeting the diagnostic criteria for ischemic stroke. Patients with cardiac, kidney, or liver failure, acquired immunodeficiency syndrome, inflammatory bowel disease and systemic infection were excluded. Morning, fasting blood samples were collected from 36 ischemic stroke patients comprising each of 18 female and male ischemic stroke subjects with matched controls. The processing and preparation of samples have been described in a previous study [39]. Briefly, samples were subjected to methanol extraction and then divided into aliquots for analysis by ultrahigh performance liquid chromatography/mass spectrometry (UHPLC/MS). The global biochemical profiling analysis was comprised of four unique arms. These were reverse phase chromatography positive ionization methods optimized for hydrophilic compounds (LC/MS Pos Polar) and hydrophobic compounds (LC/MS Pos Lipid), reverse phase chromatography with negative ionization conditions (LC/MS Neg), as well as a HILIC chromatography method coupled to negative (LC/MS Polar) [40]. These methods alternated between full-scan MS and data-dependent MSn scans. The scan range covered 70–1000 m/z with slight variation. Automated comparison of the ion features in the experimental samples to a reference library of chemical standard entries that included retention time, molecular weight (m/z), preferred adducts, and in-source fragments as well as associated MS spectra and curated by visual inspection for quality control using software developed at Metabolon was used to identify metabolites. Identification of known chemical entities was based on comparison to metabolomic library entries of purified standards [41].

### Data Analysis

Data reduction was performed using phase and baseline correction. The corrected spectra, which correspond to the chemical shift, were imported into AMIX 3.9.5 (Bruker Biospin, Rheinstetten, Germany); all spectra were reduced into integral regions with equal lengths. Regions that contained the resonance from residual water were set to zero. To reduce the concentration differences between samples, data were normalized to the total spectral area, and datasets were analyzed by pattern recognition methods using the software packages (Simca-P, version 11.5 (UmetricsAB, Umea, Sweden), and MetaboAnalyst 3.0 (www.metaboanalyst.ca, accessed on 15 September 2021). Skewed distributions for more symmetric distribution were determined by transforming the data to nonlinear conversions, while maximum variation between samples was determined using a PCA decomposition approach. This was used to determine whether the samples could be differentiated based on overarching components representing each sample’s global metabolite profile. Individual metabolites that did not have any variation between the samples were removed from the PCA analysis. The data were analyzed using a direct oblimin rotation to obtain a non-orthogonal solution. After plotting samples onto a graph based on their two components, outliers were removed from the PCA based on if they visually skewed the data. The PCA was performed for both female and male patients separately. The specific metabolites between classes were interpreted using variable importance in projection (VIP) and the correlation coefficient. A pairwise comparison was performed between the male and female stroke patients to discover distinct biomarkers associated with male and female patients among the thousands of variables using Student T-tests. The level of significance was set at *p* < 0.05. 

To select biomarker candidates for men and women patients, we used a logistic regression model. The data was split into training and validation, and the backward logistic regression model was built on the training sample set to determine the best metabolite combination. After calculating multivariate analysis, the specific metabolites between classes were selected using VIP. Metabolites with VIP scores >1.0 in the partial least squares (PLS) were examined and selected for their discrimination power by multiple statistical criteria. VIP values were used as one method of identifying biomarkers with predictive value in separating men from women. Metabolites with VIP scores ≤1 at each time point were considered irrelevant to the prediction and excluded from analysis. A similar approach was used by other studies [25,28]. The predictive ability of the model for each time point was internally validated based on leave-one-out cross validation using the Q2 diagnostic statistic. In addition, logistic regression and ROC analysis (calculated from the logistic regression) were used to establish a diagnostic model of metabolites for men and women patients. The ROC allowed us to identify metabolites that may represent candidate biomarkers for male and female AIS. The area under the ROC curve (AUC) value was used as a measure of the prognostic accuracy of individual metabolites. Therefore, while ROC curve analyses were performed for the designed model, the performance of each biomarker model was assessed using the AUC to determine sensitivity and specificity.

All variables were adjusted for in our analysis, and the effect of confounding variables did not mask our results. Just as any typical clinical studies, AIS patients who were admitted for treatment vary widely, and some related factors may confound the results of our analysis. In this study, we considered the following potentially confounding variables: gender is potentially a confounder, since the stroke incidence differs between men and women, including hospitalization rates [42]. Age at admission by admission date misused birth date as a confounder was categorized according to a related study [43]. Because comorbidities and complications associated with medication or infections were associated with AIS [20,21], and could mask the interpretation or our metabolomics results, we considered medications used including use of antibiotics, anti-hypertensive, use of cholesterol reducing medication medications, antiplatelet medications. Comorbidities’ including chronic kidney disease, atrial fibrillation disease, carotid artery stenosis, diabetes and systemic infection were considered as confounders. Treatments including the use of tissue plasminogen activator (rtPA), thrombectomy or mechanical removal of clots were added as confounding variables in our analysis. Other confounders considered in our analysis were disease status at admission and discharge including mild, severe and critical based on NIHSS stroke severity evaluation.

The metabolite profiles and pathways were analyzed for both men and women patients to reveal the metabolic network reprogramming of AIS with detailed impact using metabolomic pathways analysis of the MetaboAnalyst 3.0 software. This allowed us to determine both pathway enrichment and pathway topology, which identifies the most relevant metabolic pathways that are differentially affected in male and female ischemic stroke patients. 

## 3. Results

**Figure 1:**
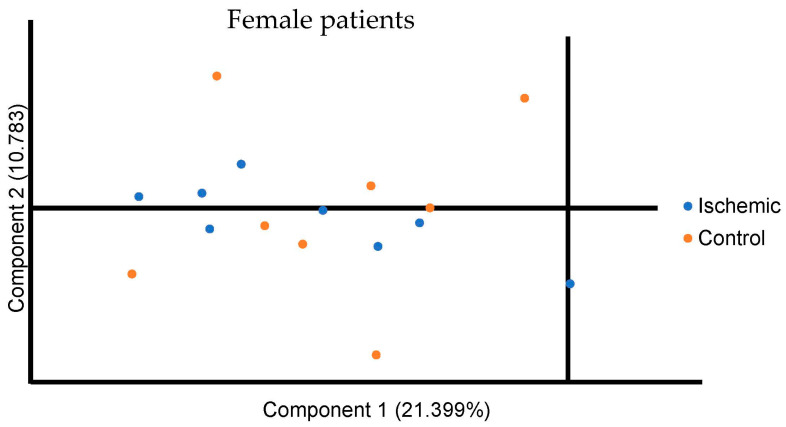
Principal Component Analysis for all metabolites in the female ischemic and control population. Component 1 is plotted on the *x* axis and Component 2 is plotted on the Y axis. Component 1 encompassed 21.399% of the variation between participants and Component 2 encompassed 10.783% of the variation between participants.

**Figure 2:**
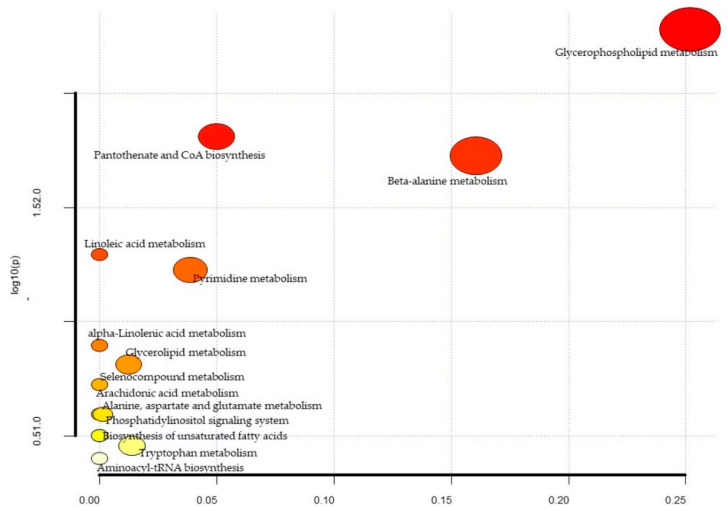
Metabolic pathway analysis for female patients. The *x*-axis represents the pathway impact, while the *y*-axis represents the −log (*p*). The analyzed pathway showed the metabolic network reprogramming of female ischemic patients with detailed impact.

A total of 1322 biochemicals, 1062 named compounds of known identity, and 260 unnamed compounds of unknown structural identity were identified. Of this, 55 metabolite levels were significantly different between the ischemic and control group in the female cohort (Table 1). The PCA did not reveal distinct potential biomarkers between the ischemic and control groups for the female population (Figure 1). As shown in the figure, component 1 encompassed 21.399% of the variance, and component 2 encompassed 10.783% of the data. The biological pathway analysis for female patients revealed fourteen different metabolic pathways (Figure 2 and Table 2), including glycerophospholipid metabolism, pantothenate, and CoA biosynthesis, beta-alanine metabolism, linoleic acid metabolism, pyrimidine metabolism, alpha-Linolenic acid metabolism, glycerolipid metabolism, seleno compound metabolism, alanine, aspartate and glutamate metabolism, phosphatidylinositol signaling system, arachidonic acid metabolism, biosynthesis of unsaturated fatty acids, tryptophan metabolism and aminoacyl-tRNA biosynthesis.

**Figure 3:**
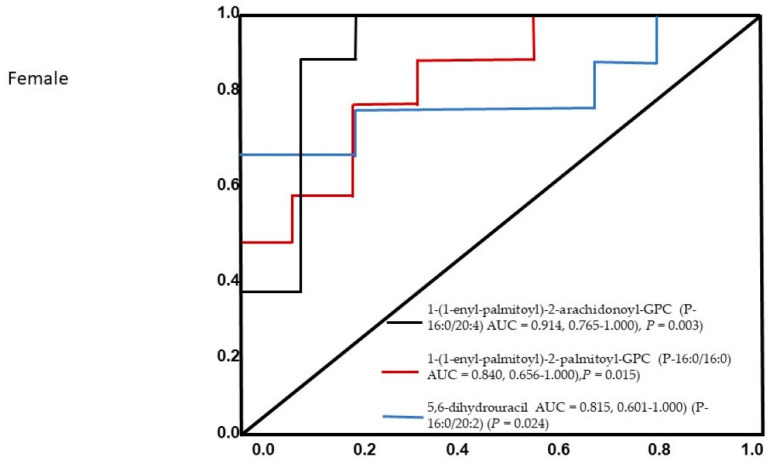
The ROC analysis results from the four diagnostic models calculated from the logistic regression analysis for female patients. The performance of each biomarker model was evaluated by the area under the ROC curve (AUC) and the determination of sensitivity and specificity at the optimal cut-off point defined by the minimum distance to the top-left corner.

Notably, 1-(1-enyl-palmitoyl)-2-arachidonoyl-GPC (P-16:0/20:4) (AUC = 0.914, 0.765–1.000), 1-(1-enyl-palmitoyl)-2-palmitoyl-GPC (P-16:0/16:0) (AUC = 0.840, 0.656–1.000), and 5,6-dihydrouracil (P-16:0/20:2) (AUC = 0.815, 0.601–1.000) were all found to be significant predictors in female ischemic stroke patients. The AUC of the optimized model was 0.945 (95% CI: 0.875–0.956) in the training set and 0.845 (95% CI: 0.721–0.923) in the validation set. None of the other metabolites were included in the model because of multicolinearity in the information provided by these compounds, and three metabolites contributed to the combined model. The constructed receiver operating characteristic (ROC) curve for the three individual metabolites is presented in Figure 3.

**Figure 4:**
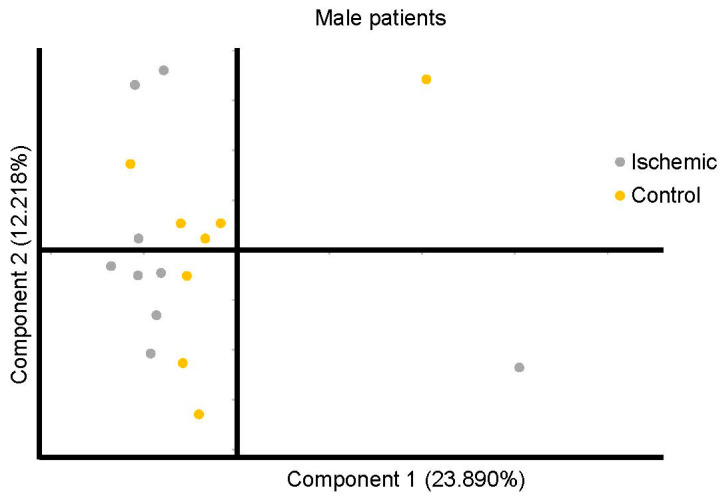
Principal Component Analysis for all metabolites in the male ischemic and control population. Component 1 is plotted on the × axis and Component 2 is plotted on the *Y* axis. Component 1 encompassed 23.890% of the variation between participants and Component 2 encompassed 12.218% of the variation between participants.

**Figure 5:**
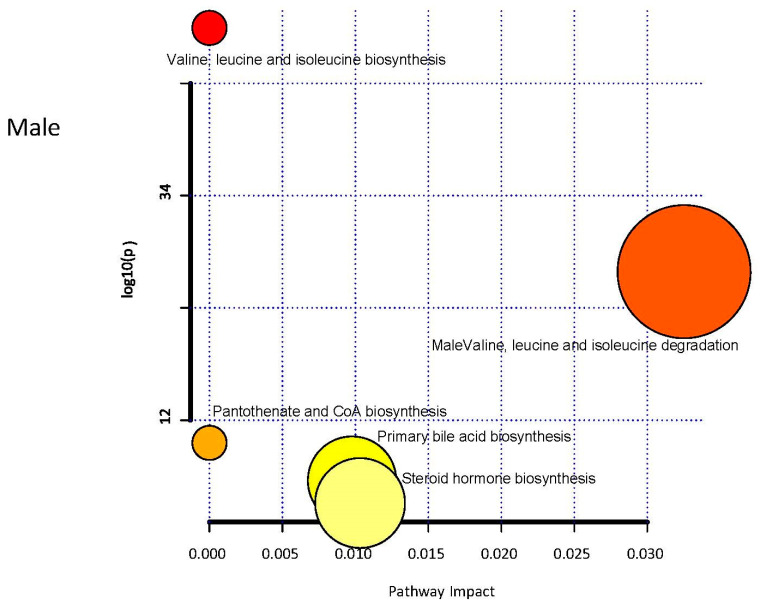
Metabolic pathway analysis for male patients. The *x*-axis represents the pathway impact, while the *y*-axis represents the −log (*p*). The analyzed pathway showed the metabolic network reprogramming of male ischemic patients with detailed impact.

**Figure 6:**
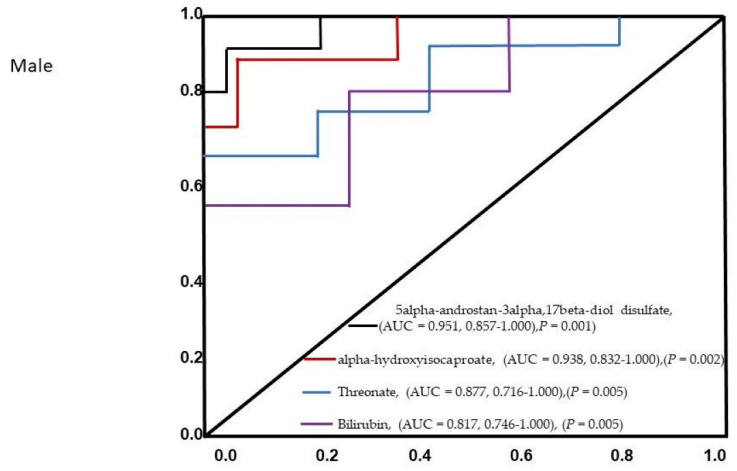
The ROC analysis results from the four diagnostic models calculated from the logistic regression analysis for male patients. The performance of each biomarker model was evaluated by the area under the ROC curve (AUC) and the determination of sensitivity and specificity at the optimal cut-off point defined by the minimum distance to the top-left corner.

The PCA did not reveal separation between male patients’ ischemic and control groups based on factor-reducing components (Figure 4). The control selection, that was not originally designed for metabolomics study may introduce selection bias, especially if not properly accounted for. This could induce the bias in the metabolite-phenotype relationships in selected groups and affect the results. Component 1 encompassed 23.890% of the variance, and Component 2 encompassed 12.218% of the data. 39 metabolite levels were significantly different between the ischemic and control group in the male cohort (Table 3). Biological pathway analysis for male patients revealed five metabolic pathways (Figure 5 and Table 4), including valine, leucine, and isoleucine biosynthesis, valine, leucine, and isoleucine degradation, pantothenate and CoA biosynthesis, primary bile acid biosynthesis, and steroid hormone biosynthesis. Significant metabolites that were predictive of male ischemic patients were 5alpha-androstan-3alpha,17beta-diol disulfate (AUC = 0.951, 0.857–1.000), alpha-hydroxyisocaproate (AUC = 0.938, 0.832–1.000), threonate (AUC = 0.877, 0.716–1.000), and bilirubin (AUC = 0.817, 0.746–1.000). The AUC of the optimized model was 0.913 (95% CI: 0.825–0.915 in the training set and 0.845 (95% CI: 0.7016–0.9015) in the validation set. The ROC curve for the four metabolites is presented in Figure 6.

## 4. Discussion

Computer tomography (CT) scans can resolve some aspects of stroke onset, and behavioral evaluations of diagnosis are entirely symptom-based, decreasing diagnostic reliability and hindering treatment [44]. Blood biomarkers related to stroke would provide an objective measurement to inform clinical assessments and treatment decisions [45]. There is no single biomarker that directly captures all the pathophysiology of stroke [45]. Therefore, using a metabolomics approach to analyze metabolites holds promise to capture the complex pathophysiological processes of AIS. This is because the metabolomics approach monitors alterations in metabolites in ways that formal identification of a single biomarker does not capture [46]. This in turn allows for the identification of metabolic changes that drive pathology.

While gender differences in risk factors of stroke has been investigated [47,48], differences in metabolites among male and female patients with AIS is not fully understood. This study uses biological samples from AIS patients from a large, carefully phenotyped epidemiologic patient cohort to identify distinctive metabolic signatures in men and women AIS patients. First, we detected 49 distinct metabolites and 15 metabolic pathways for women and 39 metabolites and 5 metabolic pathways that exhibited reprogramming. Our logistic regression and ROC analysis reveal 1-(1-enyl-palmitoyl)-2-arachidonoyl-GPC, 1-(1-enyl-palmitoyl)-2-palmitoyl-GPC, and 5,6-dihydrouracil to be significant predictors for female AIS patients. Moreover, 5alpha-androstan-3alpha,17beta-diol disulfate, alpha-hydroxyisocaproate, threonate, and bilirubin were associated with male AIS patients.

Glycerophosphocholine (GPC) metabolites modulate atherosclerosis and are associated with many risk factors [49]. GPC metabolites are platelet activating metabolites that modulate systemic oxidative stress and inflammation [50]. Findings from recent studies [51,52,53] indicate that GPC metabolites may improve the prediction of outcomes in different clinical conditions. This finding supports our current results that GPCs, including 1-(1-enyl-palmitoyl)-2-arachidonoyl-GPC and 1-(1-enyl-palmitoyl)-2-palmitoyl-GPC identified in this study, maybe sensitive indicators that can be used to help improve our understanding of the pathobiology of stroke in female patients.

Dihydrouracil (5,6-Dihydrouracil) is a metabolite of uracil that has been used as a marker to identify dihydropyrimidine dehydrogenase (DPD)-deficient [54]. Deficiencies in DPD activity are associated with reducing 5,6-Dihydrouracil catabolism, which can lead to severe toxicity in different clinical conditions [55]. Since DPD deficiency impairs the metabolic breakdown of 5,6-Dihydrouracil, the accumulation of this uracil can be detected in the plasma of patients with specific clinical conditions [56]. Therefore, identifying toxicity in stroke patients has the potential to significantly improve patient care using 5,6-Dihydrouracil as a marker. Moreover, since deficiency in enzymes downstream of DPD, such as dihydropyrimidase (DHP) and/or β-ureidopropionase (UDP), could alter the 5,6-Dihydrouracil catabolic pathway [57], our finding supports the possibility that DPD function could indicate an error of 5,6-Dihydrouracil metabolism detected in metabolomic analysis in our female ischemic stroke patients [54]. This possibility is supported by other studies [58,59] that DPD function could indicate an error of 5,6-Dihydrouracil metabolism.

5 alpha-Androstane-3 alpha 17 beta-diol is a testosterone metabolite. The modulatory role of testosterone was initially thought to be via 5 alpha reduction to the potent androgen dihydrotestosterone (DHT) and its subsequent binding to the androgen receptor [60]. However, DHT is metabolized to the estrogen receptor beta-isoform (ERbeta) agonist, 5 α-androstane 3 β, 17 β Diol (3β-Diol) [61]. This finding suggests that 3a/b-diol also represent potential precursors of DHT, and that the back conversion of DHT from 3 α—and 3 β-diol can represent a promising target in maintaining hormonal homeostasis in male ischemic stroke patients.

Alpha-hydroxyisocaproate (HICA) is derived from leucine metabolism in the human connective tissue [62]. HICA helps protect against muscle breakdown due to its anti-catabolic properties by protecting against excessive muscle damage and protein breakdown [63,64]. Elevated levels of HICA have been reported in the urine of patients with dihydrolipoyl dehydrogenase deficiency [65]. Our finding of significant levels of HICA in male AIS, suggests that HICA may be linked with ischemic stroke among male patients. In the connective tissue where HICA is well expressed, the HICA functions are not fully understood [66]. A relatively low basal protein synthesis caused by pretreatment with HICA is reported to suppress inflammatory responses of iNOS, IL-6, and ubiquitin-proteasome system’s downregulation [67]. In general, HICA is proposed to improve systemic inflammation because AMPK activation generally suppresses inflammation in several tissues [68]. The suppression of systemic inflammation results in increased energy efficiency and a decrease in proinflammatory cytokines [69]. It is also possible that systemic effects of HICA may be associated with systemic inflammation among male AIS patients. A future study on HICA among male AIS patients will help elucidate how the health benefits of HICA may contribute to the extension of healthy life in AIS patients.

Bilirubin is an end product of heme metabolism, and higher levels of serum bilirubin have been proposed to offer therapeutic advantages in oxidative stress-mediated diseases [70]. Blirubin possesses potent antioxidant properties [71], indicating that higher levels of serum bilirubin might provide a therapeutic advantage in oxidative stress-mediated conditions including stroke [72]. Mechanisms of oxidative stress-related neuronal death are associated with oxidative stress linked to pathogenesis of stroke [72]. This finding indicates that elevated serum bilirubin levels may reflect the intensity of oxidative stress [73]. In this study, we found that higher serum bilirubin was associated with male stroke patients. Therefore, male stroke patients may provide an opportunity to examine further the role of bilirubin in the pathophysiology related to oxidative stress in stroke patients. This may offer therapeutic avenues to limit the damage from a stroke facilitated by oxidative stress that results in neuronal loss.

Magnesium L-Threonate is a novel form of magnesium, and magnesium is a critical cofactor for many enzymes involved in glucose metabolism, protein production, and nucleic acid synthesis [74]. Therefore, magnesium deficiency is associated with many diseases, such as Alzheimer’s, asthma, attention deficit hyperactivity disorder, type 2 diabetes, hypertension, and stroke [75]. Moreover, magnesium intake is reported to have an inverse association with total and ischemic stroke in a dose-response pattern [76]. Our finding of an association of magnesium with male ischemic stroke patients suggests the optimization of magnesium for stroke prevention or management of stroke. This can improve not only population-wide cardiovascular health but also initiate dietary healthcare to prevent stroke. 

Several studies have investigated different metabolites in stroke [26,28,32,33]. Findings reveal new metabolites pathways for ischemic stroke subtypes and provide a new avenue to explore the pathophysiological mechanisms underlying ischemic stroke and its subtypes. A recent study by Daokun et al. [13] that focused on men and women stroke patients identified circulating biomarkers for stroke and novel pathways for AIS and its subtypes. The results reveal promising blood-based biomarkers and novel etiologic pathways of ischemic stroke, but the study did not focus on differences in metabolites between men and women AIS patients. While findings from existing studies [13,26,27,28,29,30,31,32,33,34] highlight the potential of metabolomics for discovering novel circulating biomarkers for stroke and its subtypes, most of the studies did not report gender-differentiated results. Therefore, the specific metabolites and related pathways that are directly associated with men and women AIS patients are not fully understood. As a basis for a gender-specific care for AIS patients, the characterization of differences in metabolites between the men and women AIS patients in the current study might lead to concrete starting points for a future research to improve care for men and men AIS patients.

## 5. Conclusions

The need to improve the diagnosis of stroke for both male and female patients has motivated the use of omic-based approaches to identify novel markers for stroke biology and biomarker development. Although several markers have shown promise, none has been translated into clinical practice. Ischemic stroke is a heterogeneous clinical condition and a single biomarker may not be able to capture the complicated pathophysiology changes associated with stroke in both male and female patients. In the current study, we identified (1-enyl-palmitoyl)-2-arachidonoyl-GPC, 1-(1-enyl-palmitoyl)-2-palmitoyl-GPC and 5,6-dihydrouracil as metabolites that are predictive of ischemic stroke among women while 5alpha-androstan-3alpha,17beta-diol disulfate, alpha-hydroxyisocaproate, threonate and bilirubin predicted ischemic stroke in men patients. Our findings highlight the potential of metabolomics to reveal new pathways for IS and provide a new avenue to explore IS’s pathophysiological mechanisms among men and women ischemic stroke patients.

## Figures and Tables

**Table 1:** Metabolite differences between ischemic stroke patients and control patients in the female population.

Biochemical	Super Pathway	Sub Pathway	Control	Ischemic	*p*-Value
Alanine	Amino Acid	Alanine and Aspartate Metabolism	1.29 ± 0.3	1.04 ± 0.16	0.043
Indoleacetate	Amino Acid	Tryptophan Metabolism	1.62 ± 1	0.82 ± 0.39	0.039
Isovalerate (C5)	Amino Acid	Leucine, Isoleucine and Valine Metabolism	2.11 ± 1.57	0.72 ± 0.28	0.03
3-sulfo-L-alanine	Amino Acid	Methionine, Cysteine, SAM and Taurine Metabolism	1.23 ± 0.48	0.78 ± 0.31	0.03
Retinol (Vitamin A)	Cofactors and Vitamins	Vitamin A Metabolism	1.37 ± 0.55	0.88 ± 0.29	0.037
Arachidate (20:0)	Lipid	Long Chain Saturated Fatty Acid	1.25 ± 0.26	0.97 ± 0.28	0.041
Palmitoloelycholine	Lipid	Fatty Acid Metabolism (Acyl Choline)	2.33 ± 2.23	0.54 ± 0.49	0.044
Dihomo-linolenoyl-choline	Lipid	Fatty Acid Metabolism (Acyl Choline)	4.06 ± 4.39	0.74 ± 0.77	0.04
Dtearoyl ethanolamide	Lipid	Endocannabinoid	0.88 ± 0.35	1.21 ± 0.3	0.045
N-oleoyltaurine	Lipid	Endocannabinoid	0.57 ± 0.35	1.11 ± 0.64	0.042
Glycerophosphorylcholine (GPC)	Lipid	Phospholipid Metabolism	1.43 ± 0.58	0.94 ± 0.32	0.041
1-myristoyl-2-palmitoyl-GPC (14:0/16:0)	Lipid	Phosphatidylcholine (PC)	1.92 ± 0.79	1.12 ± 0.6	0.028
1-palmitoyl-2-palmitoleoyl-GPC (16:0/16:1)	Lipid	Phosphatidylcholine (PC)	2.14 ± 1.17	1.13 ± 0.48	0.035
1-palmitoyl-2-linoleoyl-GPC (16:0/18:2)	Lipid	Phosphatidylcholine (PC)	1.17 ± 0.11	1 ± 0.14	0.014
1-palmitoyl-2-dihomo-linolenoyl-GPC (16:0/20:3n3 or 6)	Lipid	Phosphatidylcholine (PC)	1.35 ± 0.24	1 ± 0.2	0.003
1-palmitoyl-2-linoleoyl-GPE (16:0/18:2)	Lipid	Phosphatidylethanolamine (PE)	1.96 ± 0.75	1.05 ± 0.55	0.009
1-stearoyl-2-linoleoyl-GPE (18:0/18:2)	Lipid	Phosphatidylethanolamine (PE)	1.9 ± 0.77	1.14 ± 0.5	0.025
1-oleoyl-2-linoleoyl-GPE (18:1/18:2)	Lipid	Phosphatidylethanolamine (PE)	2.54 ± 1.18	1.29 ± 0.73	0.016
1-palmitoyl-2-oleoyl-GPI (16:0/18:1)	Lipid	Phosphatidylinositol (PI)	1.97 ± 0.56	1.33 ± 0.55	0.026
1-palmitoyl-2-linoleoyl-GPI (16:0/18:2)	Lipid	Phosphatidylinositol (PI)	1.83 ± 0.58	1.23 ± 0.44	0.026
1-palmitoyl-2-arachidonoyl-GPI (16:0/20:4)	Lipid	Phosphatidylinositol (PI)	1.65 ± 0.57	1.04 ± 0.36	0.016
1-linoleoyl-GPA (18:2)	Lipid	Lysophospholipid	2.2 ± 1.04	1.13 ± 0.62	0.017
1-palmitoyl-GPC (16:0)	Lipid	Lysophospholipid	1.17 ± 0.17	0.93 ± 0.11	0.003
2-palmitoyl-GPC (16:0)	Lipid	Lysophospholipid	1.28 ± 0.37	0.9 ± 0.36	0.041
1-palmitoleoyl-GPC (16:1)	Lipid	Lysophospholipid	1.79 ± 0.68	1.01 ± 0.32	0.006
2-palmitoleoyl-GPC (16:1)	Lipid	Lysophospholipid	1.63 ± 0.9	0.75 ± 0.47	0.02
1-palmitoyl-GPI (16:0)	Lipid	Lysophospholipid	1.92 ± 0.96	0.86 ± 0.64	0.015
1-stearoyl-GPI (18:0)	Lipid	Lysophospholipid	1.36 ± 0.43	0.89 ± 0.5	0.048
1-linoleoyl-GPI (18:2)	Lipid	Lysophospholipid	1.54 ± 0.59	1 ± 0.49	0.048
1-(1-enyl-palmitoyl)-2-palmitoyl-GPC (P-16:0/16:0)	Lipid	Plasmalogen	0.92 ± 0.21	1.27 ± 0.29	0.01
1-(1-enyl-palmitoyl)-2-arachidonoyl-GPC (P-16:0/20:4)	Lipid	Plasmalogen	0.86 ± 0.23	1.25 ± 0.27	0.005
1-palmitoylglycerol (16:0)	Lipid	Monoacylglycerol	2.12 ± 1.1	0.7 ± 0.31	0.004
1-palmitoleoylglycerol (16:1)	Lipid	Monoacylglycerol	2.6 ± 2.06	0.72 ± 0.44	0.026
1-oleoylglycerol (18:1)	Lipid	Monoacylglycerol	1.69 ± 1.01	0.83 ± 0.5	0.035
1-linoleoylglycerol (18:2)	Lipid	Monoacylglycerol	1.65 ± 0.92	0.85 ± 0.53	0.037
1-linolenoylglycerol (18:3)	Lipid	Monoacylglycerol	1.76 ± 1	0.86 ± 0.52	0.034
1-dihomo-linolenylglycerol (20:3)	Lipid	Monoacylglycerol	2.69 ± 2.15	0.87 ± 0.63	0.027
2-palmitoylglycerol (16:0)	Lipid	Monoacylglycerol	1.35 ± 1.11	0.39 ± 0.35	0.034
2-palmitoleoylglycerol (16:1)	Lipid	Monoacylglycerol	2.17 ± 1.99	0.31 ± 0.51	0.024
1-heptadecenoylglycerol (17:1)	Lipid	Monoacylglycerol	1.49 ± 1.04	0.61 ± 0.31	0.037
Palmitoyl-oleoyl-glycerol (16:0/18:1)	Lipid	Diacylglycerol	3.07 ± 2.59	0.95 ± 0.8	0.032
Palmitoyl-linoleoyl-glycerol (16:0/18:2)	Lipid	Diacylglycerol	1.94 ± 1.26	0.92 ± 0.53	0.046
Palmitoyl-docosahexaenoyl-glycerol (16:0/22:6)	Lipid	Diacylglycerol	1.63 ± 1.06	0.67 ± 0.46	0.03
Oleoyl-oleoyl-glycerol (18:1/18:1)	Lipid	Diacylglycerol	3.03 ± 2.4	1.09 ± 0.64	0.044
Sphingomyelin (d17:1/14:0, d16:1/15:0)	Lipid	Sphingomyelins	1.63 ± 0.52	1.13 ± 0.47	0.047
Sphingomyelin (d18:2/24:1, d18:1/24:2)	Lipid	Sphingomyelins	1.04 ± 0.2	1.27 ± 0.23	0.038
5,6-dihydrouracil	Nucleotide	Pyrimidine Metabolism, Uracil containing	0.89 ± 0.29	1.43 ± 0.6	0.027
Gamma-glutamylalanine	Peptide	Gamma-glutamyl Amino Acid	1.57 ± 0.59	0.82 ± 0.32	0.004
Gamma-glutamylhistidine	Peptide	Gamma-glutamyl Amino Acid	1.23 ± 0.37	0.81 ± 0.32	0.021
Gamma-glutamyl-epsilon-lysine	Peptide	Gamma-glutamyl Amino Acid	1.17 ± 0.29	0.9 ± 0.25	0.049
Metabolonic lactone sulfate	Partially Characterized Molecules	Partially Characterized Molecules	2 ± 1.34	0.47 ± 0.49	0.009
4-allylcatechol sulfate	Xenobiotics	Benzoate Metabolism	1.21 ± 0.79	0.52 ± 0.42	0.032
S-allylcysteine	Xenobiotics	Food Component/Plant	2.49 ± 2.47	0.32 ± 0.38	0.03
2,6-dihydroxybenzoic acid	Xenobiotics	Drug—Topical Agents	2.31 ± 1.2	1 ± 0.58	0.009
Thioproline	Xenobiotics	Chemical	1.13 ± 0.36	0.8 ± 0.27	0.048

Note: A pairwise comparison was performed between the male and female stroke patients to identify distinct biomarkers associated with male and female patients among the thousands of variables using Student *T*-tests. The level of significance was set at *p* < 0.05.

**Table 2:** A summary of the detailed results from the pathway analysis for female patients. We tested many pathways at the same time, and the statistical p values from enrichment analysis were further adjusted for multiple testings. From the table, the “Total” in the table is the total number of compounds in the pathway; the “Hits” is the actually matched number from the user uploaded data; the “Raw p” is the original p value calculated from the enrichment analysis; the “Holm p” is the p value adjusted by Holm-Bonferroni method; the “FDR p” is the p value adjusted using false discovery rate; the “Impact” is the pathway impact value calculated from pathway topology analysis.

Metabolites	Total	Expected	Hits	Raw p	−log10(p)	Holm Adjust	FDR	Impact
Glycerophospholipid metabolism	36	0.37	3	5.24 × 10^−3^	2.28 × 10^0^	4.40 × 10^−1^	4.40 × 10^1^	0.25
Pantothenate and CoA biosynthesis	19	0.20	2	1.54 × 10^−2^	1.81 × 10^0^	1.00 × 10^0^	5.24 × 10^1^	0.05
beta-Alanine metabolism	21	0.22	2	1.87 × 10^−2^	1.73 × 10^0^	1.00 × 10^0^	5.24 × 10^1^	0.16
Linoleic acid metabolism	5	0.05	1	5.06 × 10^−2^	1.30 × 10^0^	1.00 × 10^0^	9.96 × 10^1^	0.00
Pyrimidine metabolism	39	0.40	2	5.93 × 10^−2^	1.23 × 10^0^	1.00 × 10^0^	9.96 × 10^1^	0.04
alpha-Linolenic acid metabolism	13	0.13	1	1.27 × 10^−1^	8.97 × 10^−1^	1.00 × 10^0^	1.00 × 10^0^	0.00
Glycerolipid metabolism	16	0.17	1	1.54 × 10^−1^	8.13 × 10^−1^	1.00 × 10^0^	1.00 × 10^0^	0.01
Selenocompound metabolism	20	0.21	1	1.88 × 10^−1^	7.25 × 10^−1^	1.00 × 10^0^	1.00 × 10^0^	0.00
Alanine, aspartate and glutamate metabolism	28	0.29	1	2.54 × 10^−1^	5.95 × 10^−1^	1.00 × 10^0^	11.00 × 10^0^	0.00
Phosphatidylinositol signaling system	28	0.29	1	2.54 × 10^−1^	5.95 × 10^−1^	1.00 × 10^0^	1.00 × 10^0^	0.00
Arachidonic acid metabolism	36	0.37	1	3.15 × 10^−1^	5.02 × 10^−1^	1.00 × 10^0^	1.00 × 10^0^	0.00
Biosynthesis of unsaturated fatty acids	36	0.37	1	3.15 × 10^−1^	5.02 × 10^−1^	1.00 × 10^0^	1.00 × 10^0^	0.00
Tryptophan metabolism	41	0.42	1	3.50 × 10^−1^	4.56 × 10^−1^	1.00 × 10^0^	1.00 × 10^0^	0.01
Aminoacyl-tRNA biosynthesis	48	0.50	1	3.97 × 10^−1^	4.01 × 10^−1^	1.00 × 10^0^	1.00 × 10^0^	0.00

**Table 3:** Metabolite differences between ischemic stroke patients and control patients in the male population.

Biochemical.	Super Pathway	Sub Pathway	Control	Ischemic	*p*-Value
N6-acetyllysine	Amino Acid	Lysine Metabolism	1.13 ± 0.34	0.69 ± 0.21	0.004
Fructosyllysine	Amino Acid	Lysine Metabolism	0.79 ± 0.25	1.52 ± 0.93	0.037
4-methyl-2-oxopentanoate	Amino Acid	Leucine, Isoleucine and Valine Metabolism	0.8 ± 0.32	1.73 ± 1.07	0.033
Alpha-hydroxyisocaproate	Amino Acid	Leucine, Isoleucine and Valine Metabolism	0.71 ± 0.3	1.75 ± 0.68	0.001
3-methyl-2-oxovalerate	Amino Acid	Leucine, Isoleucine and Valine Metabolism	0.77 ± 0.37	1.41 ± 0.77	0.045
3-methyl-2-oxobutyrate	Amino Acid	Leucine, Isoleucine and Valine Metabolism	0.89 ± 0.25	1.36 ± 0.57	0.048
N-acetylmethionine sulfoxide	Amino Acid	Methionine, Cysteine, SAM and Taurine Metabolism	1.81 ± 1.27	0.59 ± 0.24	0.02
Threonate	Cofactors and Vitamins	Ascorbate and Aldarate Metabolism	0.58 ± 0.34	1.12 ± 0.3	0.003
Oxalate (ethanedioate)	Cofactors and Vitamins	Ascorbate and Aldarate Metabolism	0.53 ± 0.36	1.17 ± 0.42	0.003
Bilirubin (E,Z or Z,E)	Cofactors and Vitamins	Hemoglobin and Porphyrin Metabolism	1.03 ± 0.65	1.79 ± 0.85	0.049
Erucate (22:1n9)	Lipid	Long Chain Monounsaturated Fatty Acid	0.92 ± 0.28	1.2 ± 0.28	0.049
Linolenoylcarnitine (C18:3)	Lipid	Fatty Acid Metabolism (Acyl Carnitine, Polyunsaturated)	0.77 ± 0.42	1.23 ± 0.5	0.049
3-hydroxyoleoylcarnitine	Lipid	Fatty Acid Metabolism (Acyl Carnitine, Hydroxy)	0.7 ± 0.4	1.18 ± 0.54	0.047
3-hydroxydecanoate	Lipid	Fatty Acid, Monohydroxy	0.86 ± 0.38	1.39 ± 0.5	0.021
3-hydroxylaurate	Lipid	Fatty Acid, Monohydroxy	0.76 ± 0.45	1.38 ± 0.61	0.026
3-hydroxymyristate	Lipid	Fatty Acid, Monohydroxy	0.74 ± 0.31	1.41 ± 0.56	0.006
3-hydroxyoleate	Lipid	Fatty Acid, Monohydroxy	0.75 ± 0.41	1.95 ± 1.2	0.018
1-linoleoyl-GPG (18:2)	Lipid	Lysophospholipid	0.71 ± 0.35	1.18 ± 0.36	0.013
Glycosyl ceramide (d18:2/24:1, d18:1/24:2)	Lipid	Hexosylceramides (HCER)	1.2 ± 0.42	0.76 ± 0.23	0.014
5alpha-pregnan-3beta,20alpha-diol monosulfate (2)	Lipid	Progestin Steroids	0.51 ± 0.4	1.14 ± 0.71	0.039
5alpha-pregnan-3beta,20alpha-diol disulfate	Lipid	Progestin Steroids	0.78 ± 0.55	1.54 ± 0.58	0.013
Cortisone	Lipid	Corticosteroids	0.67 ± 0.43	1.13 ± 0.34	0.024
Androstenediol (3beta,17beta) monosulfate (1)	Lipid	Androgenic Steroids	0.67 ± 0.41	2.46 ± 1.87	0.021
Androstenediol (3beta,17beta) disulfate	Lipid	Androgenic Steroids	1.13 ± 0.99	2.39 ± 0.98	0.015
Androstenediol (3alpha,17alpha) monosulfate	Lipid	Androgenic Steroids	0.87 ± 0.75	2.48 ± 2.02	0.039
5alpha-androstan-3alpha,17beta-diol disulfate	Lipid	Androgenic Steroids	0.63 ± 0.39	3.06 ± 2.51	0.02
5alpha-androstan-3alpha,17beta-diol 17-glucuronide	Lipid	Androgenic Steroids	0.72 ± 0.74	1.58 ± 0.89	0.041
5alpha-androstan-3beta,17beta-diol disulfate	Lipid	Androgenic Steroids	1.26 ± 1.65	3.95 ± 2.22	0.01
Glycochenodeoxycholate	Lipid	Primary Bile Acid Metabolism	1.44 ± 0.94	0.51 ± 0.53	0.022
Glyco-beta-muricholate	Lipid	Primary Bile Acid Metabolism	1.32 ± 1.32	0.11 ± 0.06	0.026
Glycodeoxycholate	Lipid	Secondary Bile Acid Metabolism	1.93 ± 1.77	0.19 ± 0.36	0.018
Taurodeoxycholate	Lipid	Secondary Bile Acid Metabolism	1.81 ± 1.9	0.18 ± 0.16	0.033
Glycodeoxycholate 3-sulfate	Lipid	Secondary Bile Acid Metabolism	1.39 ± 0.98	0.55 ± 0.58	0.044
Gamma-glutamylphenylalanine	Peptide	Gamma-glutamyl Amino Acid	1.13 ± 0.32	0.82 ± 0.29	0.048
Gamma-glutamyltryptophan	Peptide	Gamma-glutamyl Amino Acid	1.07 ± 0.41	0.72 ± 0.22	0.04
Saccharin	Xenobiotics	Food Component/Plant	1.17 ± 1.37	0.06 ± 0	0.042
4-acetamidophenylglucuronide	Xenobiotics	Drug—Analgesics, Anesthetics	0.84 ± 0.7	0.26 ± 0.29	0.043
2-methoxyacetaminophen glucuronide	Xenobiotics	Drug—Analgesics, Anesthetics	1.09 ± 1.06	0.13 ± 0.1	0.027
3-(methylthio)acetaminophen sulfate	Xenobiotics	Drug—Analgesics, Anesthetics	1.97 ± 2.25	0.04 ± 0.07	0.033

Note: A pairwise comparison was performed between the male and female stroke patients to identify distinct biomarkers associated with male and female patients among the thousands of variables using Student *T*-tests. The level of significance was set at *p* < 0.05.

**Table 4:** Detailed results from the pathway analysis for male patients. From the table, the “Total” in the table is the total number of compounds in the pathway; the “Hits” is the actually matched number from the user uploaded data; the “Raw p” is the original p value calculated from the enrichment analysis; the “Holm p” is the p value adjusted by Holm-Bonferroni method; the “FDR p” is the p value adjusted using false discovery rate; the “Impact” is the pathway impact value calculated from pathway topology analysis.

	Total	Expected	Hits	Raw p	−log10(p)	Holm Adjust	FDR	Impact
Valine, leucine and isoleucine biosynthesis	8	0.07	3	3.20 × 10^−5^	4.49 × 10^0^	2.69 × 10^−3^	2.69 × 10^−3^	0.00
Valine, leucine and isoleucine degradation	40	0.36	3	4.76 × 10^3^	2.32 × 10^0^	3.95 × 10^−1^	2.00 × 10^−1^	0.03
Pantothenate and CoA biosynthesis	19	0.17	1	1.59 × 10^−1^	7.98 × 10^−1^	1.00 × 10^0^	1.00 × 10^0^	0.00
Primary bile acid biosynthesis	46	0.42	1	3.45 × 10^−1^	4.62 × 10^−1^	1.00 × 10^0^	1.00 × 10^0^	0.01
Steroid hormone biosynthesis	85	0.77	1	5.48 × 10^−1^	2.62 × 10^−1^	1.00 × 10^0^	1.00 × 10^0^	0.01

## Data Availability

The data presented in this study are available on request from the corresponding author.
